# New pre-treatment eosinophil-related ratios as prognostic biomarkers for survival outcomes in endometrial cancer

**DOI:** 10.1186/s12885-018-5131-x

**Published:** 2018-12-22

**Authors:** Katarzyna Holub, Albert Biete

**Affiliations:** Radiation Oncology Department, Hospital Clinic de Barcelona, University of Barcelona, C/Villarroel 170, 08036 Barcelona, Spain

**Keywords:** Endometrial cancer, Systemic inflammation, Circulating eosinophils, Neutrophil-to-lymphocyte ratio (NLR), Eosinophil-to-lymphocytes ratio (ELR), Eosinophil*neutrophil-to-lymphocytes ratio (ENLR), ESMO-ESGO-ESTRO risk assessment, Overall survival

## Abstract

**Background:**

Systemic inflammation has long been related with adverse survival outcomes in cancer patients, and its biomarkers, such as the Neutrophil-to-Lymphocyte Ratio (NLR), are recognized as poor prognostic indicators. However, the role of eosinophils in this field has been largely overlooked. Here, we describe two new pre-treatment biomarkers, expressed as Eosinophil-to-Lymphocytes Ratio (ELR) and Eosinophil*Neutrophil-to-Lymphocytes ratio (ENLR), and we analyse their impact on prognosis of endometrial cancer (EC) patients.

**Methods:**

A total of 163 consecutive patients diagnosed with EC and treated with postoperative radiotherapy +/− chemotherapy in our institution from January 2011 to December 2015 were evaluated. The cohort was divided in two groups applying the cut-off value of 0.1 and 0.5 according to ROC curve for pre-treatment ELR and ENLR, respectively. After patients’ stratification according to the ESMO-ESGO-ESTRO modified risk assessment, subgroup analyses were conducted.

**Results:**

Higher values of ELR and ENLR were associated with worse OS (*p* = 0.004 and *p* = 0.010, respectively). On univariate analysis, the factors associated with shorter OS were ELR ≥ 0.1 (HR = 2.9, *p* = 0.017), ENLR ≥ 0.5 (HR = 3.0, *p* = 0.015), advanced FIGO stage (HR = 3.4, *p* = 0.007), endometrioid histology (HR = 0.26, *p* = 0.003) and ESMO-ESGO-ESTRO high-risk (HR = 10.2, *p* = 0.023). On multivariate Cox regression, higher ELR and ENLR were independently associated with a worse outcome adjusted for the standardly applied prognostic factors.

**Conclusions:**

Increased values of ELR and ENLR portend worse OS in EC, especially in patients classified by the ESMO-ESGO-ESTRO guidelines as a high-risk group. To our best knowledge, this is the first report describing eosinophils-related ratios as prognostic biomarkers in malignant tumours.

**Electronic supplementary material:**

The online version of this article (10.1186/s12885-018-5131-x) contains supplementary material, which is available to authorized users.

## Background

Endometrial cancer (EC) is the 5th most common form of female cancer in the USA, with 61,380 new cases diagnosed in 2017, and responsible for 10,920 deaths over the same year [1]. According to the FIGO 2009 classification, 80% of EC are diagnosed in stages I-II, with 5-year survival rates of 89.6% in stage I, decreasing to 78.3% in stage II, 61.9% in stage III, and 21.1% in stage IV [2]. Although EC diagnosed in early stages is potentially curative with surgery followed by adjuvant radiotherapy +/− chemotherapy, about 15% of these patients present an increased risk of cancer progression [3]. This risk is assessed by the ESMO-ESGO-ESTRO risk stratification, based on tumour characteristics [4, 5].

However, the impact of other factors, such as systemic inflammation, is gaining importance as an indicator of poor prognostic in cancer patients [6, 7]. The interest of this host-dependent response, expressed through prognostic ratios composed of circulating white blood cells, lies mainly on circulating neutrophils, which have been studied as neutrophil-to-lymphocytes ratio (NLR) [8, 9]. Nonetheless, the role of other subpopulations of leukocytes, such as eosinophils, has been largely overlooked in tumour progression [10]. Although some reports dating from the 1950s already suggested the role of circulating eosinophils as a biomarker of tumour persistence or recurrence after radiotherapy, recent studies confirm that eosinophils act as an important modulator and effector of both innate and adaptive immune response [11–15]. Eosinophils link two different mechanisms of host defence: against allergens and against malignancies, as they secret cytokines, which guide CD8 (+) T cells and enhance their infiltration into the tumour, and induce the barrier permeability [16–19]. Recently, a high level of blood eosinophils was associated with better survival in metastatic melanoma [20].

Nonetheless, their role in cancer progression remains controversial as they may be considered as tumour-associated tissue eosinophilia (TATE) or as tumour-associated blood eosinophilia (TABE) [16, 21, 22]. Classically, TATE is more often reported in the literature and generally considered as a favourable prognosticator, while TABE is usually described as a consequence of tumour necrosis in advanced disease, hence related to poorer outcomes [23, 24]. Some authors suggest that the release of damage-associated molecular patterns (DAMPs) during tumour necrosis causes immunosuppression in tumour microenvironment and recruits diverse inflammatory cells, including circulating eosinophils, which limit the biologic activity of DAMPs [25, 26]. This mechanism may clarify why TATE has been long associated with an improved prognosis and may be the reason for the inverse association between atopic disease and the risk of cancer [27, 28].

The aim of the present study was to explore the impact of the level of pre-treatment circulating eosinophils and lymphocytes, expressed as ratios, on survival outcomes in EC patients.

## Methods

After the Institutional Review Board approval, a review of our department’s database of patients with EC treated at our institution with external beam radiotherapy (EBRT) and/or High Dose Rate Brachytherapy (HDR-BT) in a period of five years was conducted. All patients included signed the informed consent for treatment and data processing.

### Patients’ characteristics

The data of 163 patients diagnosed with histologically confirmed EC and treated with postoperative radiotherapy at our centre from January 2011 to December 2015 were retrospectively reviewed. All patients underwent hysterectomy as a first treatment, with laparoscopic pelvic paraaortic lymphadenectomy in 107 cases (81.7%), and had pre-treatment blood test results done within 3 months before surgery. Patients diagnosed with acute or chronic infections, including human immunodeficiency virus (HIV), any type of immunodeficiency, other active malignancies, haematological disorders, steroid or anti-inflammatory treatment for any reason, were excluded. The median age at diagnosis was 65 years (y), mean 64.79, range 41–90. The most frequent histology was endometrioid carcinoma (73.3%). The disease stage was classified as non-advanced (stage I-II) in 98 patients (74.8%).

The combination of EBRT and HDR-BT was administered to 88 patients (65.2%), with a mean dose of 53.4 Gray (Gy) (range 21–75). Only 43 patients (32.8%) were treated exclusively with HDR-BT (mean dose 10.0 Gy, range 7–20). All patients were stratified into six risk groups according to ESMO-ESGO-ESTRO anatomopathological features [4]. Patients’ characteristics are detailed in Table 1.

**Table 1:** Characteristics of all patients included in the study (*n* = 163), comparison of these characteristics according to ELR (cut-off ≥ 0.1) and ENLR (cut-off ≥ 0.5)

Patients’ characteristics	All patients included (*n* = 163, % of total)	ELR < 0.1 *n* = 117 (%)	ELR ≥ 0.1 *n* = 46 (%)	*p*-value*	ENLR < 0.5 *n* = 118 (%)	ENLR ≥0.5 *n* = 45 (%)	*p*-value*
Age at diagnosis (years):							
< 65 years:	74 (45.4%)	4 (73.0%)	20 (27.0)	.757	53 (71.6%)	21 (28.4%)	.410
≥ 65 years:	89 (54.6%)	63 (70.8%)	26 (29.2)		65 (73.0%)	24 (27.0%)	
FIGO 2009 stage at diagnosis:							
• IA	4 (26.0%)	34 (7.1%)	9 (0.9%)	.070	33 (76.7%)	10 (23.3%)	.136
• IB	54 (33.2%)	33 (61.1%)	21(38.9%)		35 64.8%)	19 (35.2%)	
• II	24 (14.0%)	19 (79.2%)	5 (20.8%)		18 (75.0%)	6 (25.0%)	
• IIIA	9 (5.5%)	4 (44.4%)	5 (55.6%)		5 (55.6%)	4 (44.4%)	
• IIIB	0	0	0		0	0	
• IIIC1	15 (9.2%)	13 (86.7%)	2 (13.3%)		14 (93.3%)	1 (6.7%)	
• IIIC2	7 (4.3%)	7(100%)	0		7 (100%)	0	
• IVA	9 (5.5%)	6 (66.7%)	3 (33.3%)		5 (55.6%)	4 (44.4%)	
• IVB	2 (1.2%)	1 (50%)	1 (50%)		1 (50.0%)	1 (50.0%)	
Patients in advanced stage (FIGO I-II vs. FIGO III-IV) (n,%):							
No (FIGO I-II)	121(74.2%)	85 (70.2%)	36(29.8%)	.461	85 (70.2%)	36 (29.8%)	.299
Yes (FIGO III-IV)	42 (25.8%)	32 (76.2%)	10(23.8%)		33 (78.6%)	9 (21.4%)	
Tumour grade (n,%):							
1	36 (22.1%)	24 (66.7%)	12(33.3%)	.736	26 (72.2%)	10 (27.8%)	.772
2	61 (37.4%)	45 (73.8%)	16(26.2%)		46 (75.4%)	15 (24.6%)	
3	66 (40.5%)	48 (72.7%)	18(27.3%)		46 (69.7%)	20 (30.3%)	
Patients with tumour grade 3 vs. tumour grade 1–2 (n,%):							
Grade 1–2	97 (59.5%)	66 (68.0%)	31(32.0%)	.865	72 (74.2%)	25 (25.8%)	.593
Grade 3	66 (40.5%)	46 (69.7%)	20(30.3%)		46 (69.7%)	20 (30.3%)	
Tumour histology (n,%):							
Endometrioid	119 (73%)	85 (71.4%)	34(28.6%)	.305	87 (73.1%)	32 (26.9%)	.424
Serous-papillary	14 (8.6%)	12 (85.7%)	2 (14.3%)		10 (71.4%)	4 (28.6%)	
Clear cell	5 (3.1%)	5 (100%)	0		5 (100%)	0	
Squamous	6 (3.7%)	3 (50.0%)	3 (50.0%)		3 (50.0%)	3 (50.0%)	
Villoglandular	2 (1.2%)	1 (50.0%)	1 (50.0%)		1 (50.0%)	1 (50.0%)	
Sarcoma (not LMS or carcinosarcoma)	2 (1.2%)	2 (100%)	0		2 (100%)	0	
Leiomyosarcoma	3 (1.8%)	1 (33.3%)	2 (66.7%)		1 (33.3%)	2 (66.7%)	
Carcinosarcoma	12 (7.4%)	8 (66.7%)	4 (33.3%)		9 (75.0%)	3 (25.0%)	
Lymphadenectomy (n,%):							
No	29 (17.8%)	19 (65.6%)	10(34.5%)	.409	19 (65.5%)	10 (34.5%)	.361
Yes	134 (82.2%)	98 (73.1%)	36(26.9%)		99 (73.9%)	35 (26.1%)	
ESMO-ESGO-ESTRO (ESMO 2015) risk groups (n,%):							
1 - Low	28 (17.2%)	21 (75.0%)	7 (25.0%)	.775	21 (75.0%)	7 (25.0%)	.440
2 - Intermediate	31 (19.0%)	20 (64.5%)	11(35.5%)		21 (67.7%)	10 (32.3%)	
3- High-Intermediate	13 (8.0%)	9 (69.2%)	4 (30.8%)		8 (61.5%)	5 (38.5%)	
4 - High	75 (46.0%)	57 (76.0%)	18(24.0%)		59 (78.7%)	16 (21.3%)	
5 - Advanced	14 (8.6%)	9 (64.3%)	5 (35.7%)		8 (57.1%)	6 (42.9%)	
6 - Metastatic	2 (1.2%)	1 (50.0%)	1 (50.0%)		1 (50.0%)	1 (50.0%)	
Patients in high-risk groups according to ESMO-ESGO-ESTRO risk classification (n,%):							
Low (risk group 1–3)	72 (44.2%)	47 (65.3%)	25(34.7%)	.496	50 (69.4%)	22 (30.6%)	.484
High (risk group 4–6)	91 (55.8%)	65 (71.4%)	26(28.6%)		68 (74.7%)	23 (25.3%)	
Brachytherapy exclusive (n,%):							
No	110 (67.5%)	75 (68.2%)	35(31.8%)	.142	77 (70.0%)	33 (30.0%)	.325
Yes	53 (32.5%)	42 (79.2%)	11(20.8%)		41 (77.4%)	12 (22.6%)	
Chemotherapy (n,%):							
No	104 (63.8%)	72 (69.2%)	32(30.8%)	.337	71 (68.3%)	33 (31.7%)	.118
Yes	59 (36.2%)	45 (76.3%)	14(23.7%)		47 (79.7%)	12 (20.3%)	

* *X2 test* or *T-student test*

### Follow-up results

The follow up (FU) was performed, as suggested by ESMO guidelines, every 3–4 months for the first 2 years, and then every 6 months for the next 3 years.

### Systemic inflammation biomarkers

The level of eosinophils in pre-treatment blood tests was analysed. We created two groups using a 0.1 cut-off according to the best receiver operating characteristics (ROC) curve value: patients with absolute eosinophil count (AEC) ≥ 0.1 (*n* = 112) vs. AEC <  0.1 (*n* = 19), and we evaluated the influence of high AEC on patients’ survival outcomes.

Afterwards, we described two new systemic inflammatory biomarkers, expressed as follows: Eosinophil-to-Lymphocytes Ratio (ELR) and Eosinophil*Neutrophil-to-Lymphocytes Ratio (ELR multiplied by the absolute neutrophil count, ENLR), and we analysed their impact on overall survival (OS) and progression free survival (PFS). Additionally, we evaluated the same ratios based on post-treatment blood tests.

### Statistical analysis

All statistical tests were two-sided and statistical significance was defined as *p* <  0.05. Summarized data are presented as numbers and percentages unless otherwise stated.

The primary endpoint was OS and the secondary endpoint was PFS. Predefined subgroup analysis was conducted based on the ESMO-ESGO-ESTRO risk stratification, which subsequently was converted into a binary variable by creating a low (groups 1–3) and a high-risk cohort (groups 4–5).

Afterwards, the study population was subdivided into two groups, based on the cut-offs for ELR and ENLR, separately. Both ELR and ENLR were defined as binary variables by finding the cut-off value from a ROC curve. The binary variables’ balance across prognostic characteristics was assessed using Chi-square test (X2 test). Frequencies were compared using Fisher’s exact test for categorical variables. Kaplan Meier’s curves were displayed to evaluate the prognostic value of ELR and ENLR for OS and PFS. Survival outcomes were calculated from the date of surgery to the event occurrence, which is progression or cancer-related death in the case of PFS, or any death in the case of OS. If no event occurred, patients were censored at the time they were last known to be event free. All survival outcomes were analysed using Log rank test (LR), Breslow test (BR) was additionally applied.

The univariate and multivariate Cox regression models were used to assess the prognostic effect of inflammatory biomarkers and included binary variables: ELR (≥ 0.1 vs. < 0.1), ENLR (≥ 0.5 vs. < 0.5), age (≥ 65y vs. < 65y), ESMO-ESGO-ESTRO high-risk (groups 4–6) vs. low-risk (groups 1–3), high grade (grade 3 vs. grade 1–2), advanced FIGO 2009 stage (stage III-IV vs. stage I-II), endometrioid histology (vs. other tumour histology). All statistical analyses were performed using SPSS v. 23.

## Results

### Survival outcomes of the entire cohort

All survival outcomes were expressed in months. After a median follow-up of 54.8 (range 24.6–58.4), progression was observed in 36 patients (22.1%). There was no exclusive local recurrence and only one patient developed exclusive regional recurrence (progression in pelvic node). Both local and regional recurrence, were observed in 10.7% of patients (*n* = 14), while 17.6% (*n* = 23) presented distant metastasis. Median PFS for the entire cohort was of 23.1 (range 0.2–62.2). At the moment of data collection, 21 deaths were reported (12.33%), 20 of them were related to cancer (12.27%). All details concerning the survival outcomes according to the ESMO-ESGO-ESTRO risk groups are included in Table 2.

**Table 2:** Patients’s status at the end of the study according to ESMO-ESGO-ESTRO modified risk assessment (ESMO 2015)

ESMO 2015 risk group	Status of patients at the end of the study (n = 163)				
	Alive (no evidence of disease)	Disease progression	Cancer-related death	Non cancer related death	Total
1	27 (20.8%)	1 (8.33%)	0	0	28 (17.2%)
2	30 (23.1%)	0	0	1 (100%)	31 (19.0%)
3	11 (8.4%)	0	2 (10%)	0	13 (8.0%)
4	57 (43.8%)	7 (58.33%)	11 (55%)	0	75 (46.0%)
5	5 (3.9%)	4 (33.33)	5 (25%)	0	14 (8.6%)
6	0	0	2 (10%)	0	2 (1.2%)
Total	130 (100%)	12 (100%)	20 (100%)	1 (100%)	163 (100%)

### ELR

The mean value of ELR was 0.08 (SD 0.065, range 0.0–0.31, median 0.063). Using the cut-off of 0.1 according to the ROC with the Area Under Curve (AUC) of 0.61, we divided the entire cohort into two groups: ELR ≥ 0.1 (*n* = 46) and ELR <  0.1 (*n* = 117). Patients’ characteristics of the two comparative groups are included in Table 1.

**Fig. 1:**
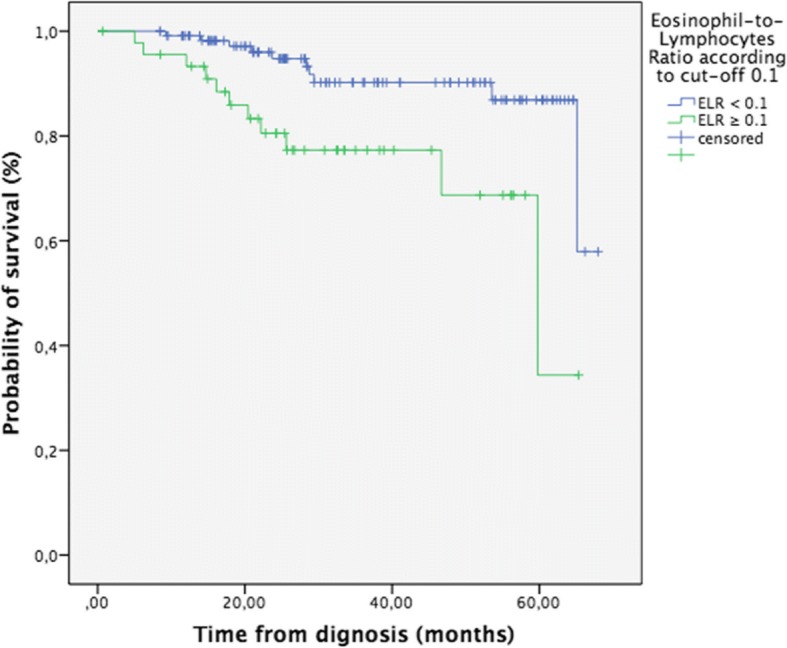
Overall survival of patients with endometrial cancer (*n* = 163) according to the Eosinophil-to-Lymphocytes Ratio (ELR, cut-off ≥ 0.1), *p* = 0.004

OS in the group with ELR ≥ 0.1 was 50.7 months (IC 95% 43.8–57.6) vs. 62.3 months (IC 95% 59.0–65.5) in the group with ELR <  0.1 (LR *p* = 0.004, X2 = 6.3; BR *p* = 0.026, X2 = 4.9), HR 2.9, *p* = 0.017 (Fig. 1).

Eleven events were reported in the high ELR group (23.9% of patients) vs. 10 events (8.6%) in the low ELR group (LR: p = 0.01, X2 = 6.5; BR: p = 0.026, X2 = 4.9).

Regarding the data of the entire cohort, PFS of patients according to ELR level was not significantly different (LR *p* = 0.095, BR *p* = 0.08). However, the number of events was higher in patients with ELR ≥ 0.1 (14 events vs. 22 events, which meant 30.4% vs. 18.8% patients with progression, respectively).

ELR was not correlated with patient’s age (*p* = 0.90), FIGO stage at diagnosis (*p* = 0.77), tumour histology (*p* = 0.94) or tumour grade (*p* = 0.86).

### ENLR

The mean value of ENLR was 0.448 (SD 0.59, range 0–5.54, median 0.31). Using the cut-off of 0.5 (ROC curve AUC = 0.621), we divided the entire cohort into two groups: ENLR ≥0.5 (*n* = 45) and ENLR < 0.5 (*n* = 118). Patients’ characteristics of the two comparative groups are included in Table 1.

**Fig. 2:**
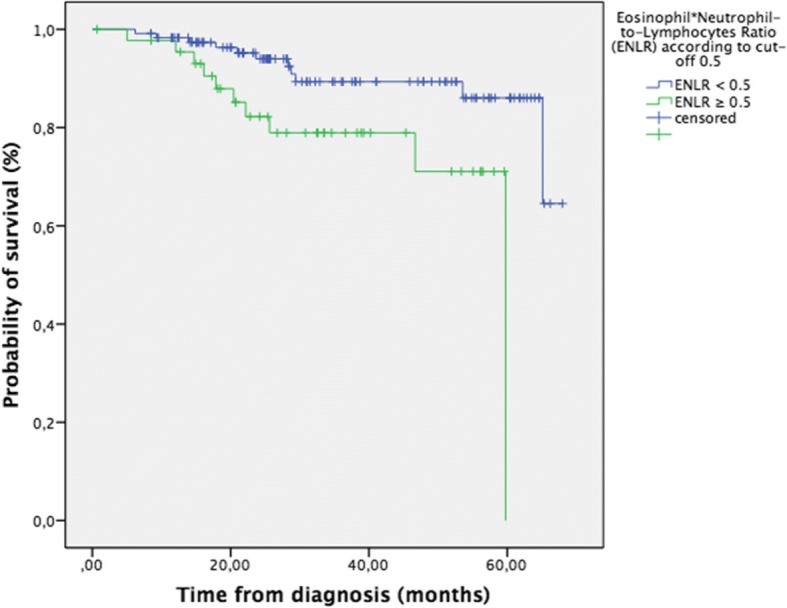
Overall survival of patients with endometrial cancer (*n* = 163) according to the Eosinophil*Neutrophil-to-Lymphocytes Ratio (ENLR, cut-off  ≥ 0.5), *p* = 0.010

Median OS in the group with ENLR ≥ 0.5 was 49.8 months (IC 95% 43.8–55.8) vs. 61.9 months (IC 95% 58.6–65.2) in the group with ENLR < 0.5 (LR: *p* = 0.01, X2 = 6.6; BR: *p* = 0.026, X2 = 4.9, HR = 3.0, *p* = 0.015, Fig. 2). Ten events were reported in the high ENLR group (which meant 22.2% of patients) vs. 11 events (9.3%) in the low ENLR group.

Median PFS according to ENLR level was not significantly different (LR: *p* = 0.1, BR: *p* = 0.09).

ENLR < 0.5 was not correlated with patients’ age (*p* = 0.81), FIGO stage at diagnosis (*p* = 0.22), tumour histology (*p* = 0.63) or tumour grade (*p* = 0.59).

### ELR and ENLR as independent prognostic factors

On univariate analysis, worse OS was associated with ELR ≥ 0.1 (HR = 2.9, *p* = 0.017), ENLR ≥ 0.5 (HR 3.0, *p* = 0.015), ESMO-ESGO-ESTRO high-risk (HR = 4.7, *p* = 0.014), tumour grade 3 (HR = 8.1, *p* = 0.001) and advanced stage (HR = 3.4, *p* = 0.007), while endometrioid histology was associated with better prognosis (HR = 0.3, *p* = 0.003). Age ≥ 65 years did not reach significance, *p* = 0.055 (Table 3). All variables included in univariate analysis were evaluated in all patients using X2 tests (Table 1, Additional file 1: Figure S1, Additional file 2: Figure S2, Additional file 3: Figure S3, Additional file 4: Figure S4, and Additional file 5: Figure S5).

**Table 3:** Impact of ELR and ENLR on overall survival (OS): Kaplan Meier survival analysis, univariate and multivariate Cox regression (models for ELR and ENLR, respectively), *n* = 163

Variables	Kaplan-Meier survival analysis		UNIVARIATE Cox regression		MULTIVARIATE Cox regression for ELR ≥ 0.1 model		MULTIVARIATE Cox regression for ENLR ≥0.5 model	
	X2	*p*-value	HR (IC 95%)	*p*-value	HR (IC 95%)	*p*-value	HR (IC 95%)	*p*-value
Age ≥ 65 years (vs. < 65 years)	3.9	**0.047**	2.6 (0.98–6.7)	0.055	2.8 (1.0–8.2)	0.059	2.8 (1.03–7.8)	**0.043**
FIGO advanced stage III (vs. stage I-II)	8.3	**0.004**	3.4 (1.4–8.4)	**0.007**	3.1 (0.9–9.8)	0.060	2.8 (0.9–8.5)	0.065
Tumour grade 3 (vs. grade 1–2)	15.8	**0.000**	8.1 (2.4–27.4)	**0.001**	6.8 (1.9–24.7)	**0.003**	5.8 (1.6–21.2)	**0.007**
Endometroid histology (vs. other histology)	10.1	**0.001**	0.3 (0.1–0.63)	**0.003**	0.4 (0.1–1.1)	0.068	0.4 (0.14–1.2)	0.099
ESMO-ESTRO-ESGO High Risk groups 4–6 (vs. groups 1–3)	7.4	**0.007**	4.7 (1.4–15.8)	**0.014**	1.2 (0.2–6.5)	0.856	1.3 (0.2–6.9)	0.770
ELR ≥ 0.1 (vs. < 0.1)	6.3	**0.004**	2.9 (1.2–6.8)	**0.017**	4.9 (1.9–12.4)	**0.001**	–	–
ENLR ≥ 0.5 (vs. < 0.5)	6.6	**0.010**	3.0 (1.2–7.3)	**0.015**	–	–	3.9 (1.6–9.8)	**0.003**

All entries in boldface reflect *p*-values < 0.05

On multivariate analysis, carried out separately for ELR and ENLR, the impact of variables used in univariate analysis on the principal endpoint (OS) was evaluated. Three variables reached statistical significance: ELR ≥ 0.1 (HR = 4.9, *p* = 0.001), ENLR ≥0.5 (HR = 3.9, *p* = 0.003), and tumour grade 3 (*p* = 0.003 in ELR model and *p* = 0.007 in ENLR model). Age ≥ 65 was statistically significant only in ENLR model (*p* = 0.043) (Table 3).

### Subgroups according to ESMO-ESGO-ESTRO modified risk assessment

According to the ESMO-ESGO-ESTRO risk classification, all patients were allocated to six different risk groups (Table 1). Thus, we divided the entire cohort into two groups: low-risk (groups 1–3) and high-risk cohort (groups 4–6).

In the high-risk cohort (*n* = 91), 34.6% of patients with ELR ≥ 0.1 died and OS was of 44.9 months (IC 95% 35.9–53.9) vs. 59.6 months (54.7–64.5) in the low ELR group with 13.8% of patients dead at the end of the study (LR: *p* = 0.009, X2 = 6.8, BR: *p* = 0.015, X2 = 5.9, Additional file 6: Figure S6). PFS was not significantly different between the two groups (*p* = 0.10).

Patients in the ENLR ≥ 0.5 cohort showed a worse OS of 45.5 months (IC 95%, 36.2–54.9) vs. 59.0 months in ENLR < 0.5 (IC 95%, 54.1–64.0, LR: *p* = 0.020, X2 = 5.4, BR: *p* = 0.047, X2 = 3.9, Additional file 7: Figure S7). PFS was not significantly different between the two groups (*p* = 0.18).

In the low-risk cohort (*n* = 72), patients with ELR ≥ 0.1 (*n* = 25) and patients with ENLR ≥ 0.5 (*n* = 22) failed to show statistically significant differences regarding OS and PFS.

### Absolute eosinophil count (AEC)

Pre-treatment blood eosinophilia, defined as an absolute eosinophils count ≥ 0.65 × 10^9^/L, was detected in our cohort only in 6 patients (4.6%), all of them alive and with no evidence of disease progression at the moment of data collection. The mean value of AEC was 0.1 (SD 0.13, range 0–0.8, median 0.152). There were no statistical differences in OS (55.6 months vs. 61.35 months, *p* = 0.154, X2 = 2.04) nor PFS (*p* = 0.772, X2 = 0.08) between the group with AEC ≥ 0.1 and the one with AEC < 0.1.

### Leucocytosis, neutrophilia and NLR

Patients that presented a high level of circulating WBC (leukocytes > 11,000 × 10^9^/L or neutrophils > 7000 × 10^9^/L) or NLR ≥ 2.4 (cut-off according to ROC curve, AUC 0.516) at cancer diagnosis did not show statistical difference in OS compared to patients with lower levels (*p* = 0.51, *p* = 0.23 and *p* = 0.63, respectively).

## Discussion

Systemic inflammation is a recognised feature of cancer progression, and inflammatory biomarkers are a key subject of research on anti-tumour response. However, the role of eosinophils in this field has long been ignored. Even though tumour-associated blood eosinophilia is described in a wide range of tumours [11, 12, 27], and is easily diagnosed, this finding is not so frequent in clinical practice, and only accounts for 1–7% of all eosinophilia’s diagnoses [21, 29]. In accordance with these reports, we concluded that eosinophilia at cancer diagnosis was really infrequent in our cohort and had no impact on survival outcome. We hypothesise that it is not eosinophils alone, but the ratios between circulating eosinophils and lymphocytes, that may reflect the host’s immunosuppression status at cancer diagnosis, and may help to achieve a more precise risk stratification of patients diagnosed with EC.

Here, we describe new eosinophil-based prognostic ratios, expressed as a relationship between different subtypes of WBC. To our best knowledge, we are the first to propose these ratios as prognostic biomarkers in malignant tumours and to apply inflammatory biomarkers as a tool to refine the ESMO-ESGO-ESTRO risk stratification in EC.

We focused our study on EC because, in spite of its generally favourable prognosis, recent studies claim that the survival outcomes depend on factors beyond the classically established risk indicators [30]. Moreover, the scientific evidence of the inflammatory biomarkers in EC is significantly lower than in other malignancies [7–9].

Our study is based on a uniformly treated cohort that includes patients of all FIGO stages if they underwent hysterectomy as a first treatment. According to the ESMO guidelines, a complete macroscopic cytoreduction is recommended even for advanced disease, while systematic pelvic lymphadenectomy should not be performed routinely, hence lymphadenectomy was performed only in 80% of patients in our study [4]. Most patients with EC usually present a low recurrence risk, but in our study 55.8% of patients (*n* = 91) belonged to the high-risk group, which may be explained by the reference status of our institution, where patients with high-risk factors are usually addressed. Although distant relapses often account for only one-third of recurrences in the literature, they were observed in 63.9% of all relapses in our cohort [4, 30]. As most EC relapses occur within 3 years after the primary treatment, we considered that our median FU of 55.9 months (range 52.4–59.4) was long enough [4].

Due to the correlation between ELR and ENLR, two multivariate analysis models were evaluated: one for ELR ≥ 0.1 (*p* = 0.001, HR = 4.9, IC 95%, 1.9–12.4) and one for ENLR ≥ 0.5 (*p* = 0.003, HR = 3.9, IC 95%, 1.6–9.8). Holding the other covariates constant, higher values of ELR and ENLR were strongly associated with an increased risk of death and were independent indicators of poorer overall survival. Contrarily, ratios based on post-treatment blood tests had no impact on patients’ prognosis.

Similarly, pre-treatment ELR and ENLR showed an impact on OS and PFS in the high-risk group, but not in the low-risk group (*p* = 0.21 and *p* = 0.18, respectively), in which a limited number of events was observed.

On univariate analysis of the entire cohort, the variable age ≥ 65y only trended toward significance (*p* = 0.055) but was maintained in multivariate analysis as a clinically important factor, and was significantly associated with worse OS in ENLR Cox model. In the high-risk patients (groups 4–6), age ≥ 65y was an indicator of poor survival (*p* = 0.014 in univariate analysis) and was proven to be an independent prognostic factor in both Cox regression models [Table 4]. By contrast, FIGO advanced stage and endometrioid histology were not significant prognosticators in the univariate analysis of high-risk patients.

**Table 4:** Impact of ELR and ENLR on overall survival (OS): Kaplan-Meier survival analysis, univariate and multivariate Cox regression (models for ELR and ENLR, respectively) in high-risk patients (ESMO-ESGO-ESTRO groups 4–6, *n* = 91)

Variables	Kaplan-Meier survival analysis		UNIVARIATE Cox regression		MULTIVARIATE Cox regression for ELR ≥ 0.1 model		MULTIVARIATE Cox regression for ENLR ≥0.5 model	
	X2	*p*-value	HR (IC 95%)	*p*-value	HR (IC 95%)	*p*-value	HR (IC 95%)	*p*-value
Age ≥ 65 years (vs. < 65 years)	6.9	**0.008**	3.7 (1.3–10.5)	**0.014**	3.2 (1.03–10.3)	**0.045**	3.2 (1.1–9.7)	**0.038**
FIGO advanced stage III (vs. stage I-II)	2.2	0.135	2.1 (0.8–5.7)	0.143	2.6 (0.8–8.6)	0.109	2.5 (0.8–7.7)	0.104
Tumour grade 3 (vs. grade 1–2)	6.7	**0.010**	5.6 (1.3–24.5)	**0.022**	6.9 (1.5–31.6)	**0.013**	5.8 (1.3–26.3)	**0.023**
Endometroid histology (vs. other histology)	1.7	0.194	0.5 (0.2–1.4)	0.202	0.5 (0.2–1.5)	0.195	0.5 (0.2–1.6)	0.257
ELR ≥ 0.1 (vs. < 0.1)	3.9	**0.047**	3.3 (1.3–8.7)	**0.014**	4.6 (1.7–12.5)	**0.002**	–	–
ENLR ≥0.5 (vs. < 0.5)	5.4	**0.020**	3.0 (1.1–7.7)	**0.026**	–	–	3.9 (1.6–9.8)	**0.011**

All entries in boldface reflect *p*-values < 0.05

ENLR was described in order to demonstrate the importance of the relation between eosinophils and lymphocytes. Subsequently, we observed that the impact on OS was constant and not influenced by the neutrophil count. As both eosinophil-based ratios have proved to be independent prognostic factors for OS, while NLR has not, we concluded that the relation between eosinophils and lymphocytes was not affected by the presence of neutrophils, which may be interpreted as a superiority of eosinophil-based ratios over NLR and Platelet-to-Lymphocytes Ratio (PLR) (Additional file 8: Figure S8).

Our study presents some limitations, being a retrospective single institution cohort with a relatively small number of patients, which may produce potential confounding biases. However, almost all studies that deal with systemic inflammation biomarkers are of retrospective nature [7–9, 29, 30]. Confirming the cut-off points for ELR and ENLR in a larger cohort, preferably in a multicentre study, would be important for any future investigation.

In our opinion, the most important potential bias in the application of the biomarkers of systemic inflammation in clinical practice is the short life span of the WBC in systemic circulation. Consequently, it is difficult to completely rule out a potential influence of some temporary acute immunological changes, such as asymptomatic infections, on the systemic inflammatory response.

We are convinced that our investigation may contribute to a new stratification of EC and to further immunotherapy research that aim at the eosinophilic-mediate anti-tumour response.

## Conclusions

Our study presents a new concept of the role of eosinophils in cancer progression that may be used as a novel prognostic tool for EC stratification. Increased values of eosinophil-related ratios based on pre-treatment blood tests are associated with worse OS in all EC patients and in high-risk patients of the ESMO-ESGO-ESTRO modified risk assessment. Impact of ELR and ENLR on PFS did not achieve statistical significance.

According to these results, the described ratios are of interest for EC prognosis and should be considered in the pre-treatment analysis. To our best knowledge, this is the first report that describes and analyses eosinophil-related ratios as prognostic indicators in cancer patients.

## Additional files

Additional file 1: **Figure S1.** Overall survival according to FIGO stage (*n* = 163). Kaplan-Meier survival analysis (*p* = 0.004 Log Rank, *p* = 0.013 Breslow test). (DOCX 64 kb)

Additional file 2: **Figure S2.** Overall survival according to tumour grade (*n* = 163). Kaplan-Meier survival analysis (*p* = 0.001 Log Rank, *p* = 0.023 Breslow test). (DOCX 70 kb)

Additional file 3: **Figure S3.** Overall survival according to ESMO-ESGO-ESTRO risk assessment (*n* = 163): high-risk (groups 1–3) vs. low-risk (groups 4–6). Kaplan-Meier survival analysis (*p* = 0.007 Log Rank, *p* = 0.005 Breslow test). (DOCX 77 kb)

Additional file 4: **Figure S4.** Overall survival according to tumour histology (n = 163): endometrioid vs. non endometrioid (*p* = 0.001 Log Rank, *p* = 0.004 Breslow test). (DOCX 66 kb)

Additional file 5: **Figure S5.** Overall survival according to patients’ age at diagnosis (n = 163). Kaplan-Meier survival analysis (*p* = 0.047 Log Rank, *p* = 0.036 Breslow test). (DOCX 70 kb)

Additional file 6: **Figure S6.** Overall survival of patients in High Risk (risk groups 4–6 of ESMO 2015 classification) stratified by Eosinophil-to-Lymphocytes Ratio (ELR) according to cut-off 0.1. (DOCX 65 kb)

Additional file 7: **Figure S7.** Overall survival of patients in High Risk (Risk groups 4–6 of ESMO 2015 classification) stratified by Eosinophil*Neutrophil-to-Lymphocytes Ratio (ENLR) according to cut-off 0.5. (DOCX 65 kb)

Additional file 8: **Figure S8.** ROC curves for ELR, ENLR, NLR and Platelet–to-Lymphocytes Ratio (PLR). (DOCX 81 kb)

## Abbreviations

AUC: Area Under Curve
BR: Breslow test
DAMPs: Damage-associated molecular patterns
EBRT: External Beam Radiotherapy
EC: Endometrial Cancer
ELR: Eosinophil-to-Lymphocytes Ratio
ENLR: Eosinophil*Neutrophil-to-Lymphocytes Ratio
ESMO-ESGO-ESTRO: European Society for Medical Oncology, European Society for Radiotherapy & Oncology, European Society of Gynaecological Oncology
FIGO: International Federation of Gynaecology and Obstetrics
Gy: Gray
HDR-BT: High Dose Rate Brachytherapy
HIV: Human Immunodeficiency Virus
HR: Hazard Ratio
LR: Log rank test
NLR: Neutrophil-to-Lymphocytes ratio
OS: Overall Survival
PFS: Progression Free Survival
TABE: Tumour-associated blood eosinophilia
TATE: Tumour-associated tissue eosinophilia
X2: Chi-square test
y: Years
