# Supplementary figures and images for: New pre-treatment eosinophil-related ratios as prognostic biomarkers for survival outcomes in endometrial cancer
Source: BMC Cancer. 2018 Dec 22;18:1280. doi: 10.1186/s12885-018-5131-x (PMC6304088; doi:10.1186/s12885-018-5131-x)

Supplementary Figure 1. Overall survival according to FIGO stage (n=163), p=0.004 Log Rank, p= 0.013 Breslow test.


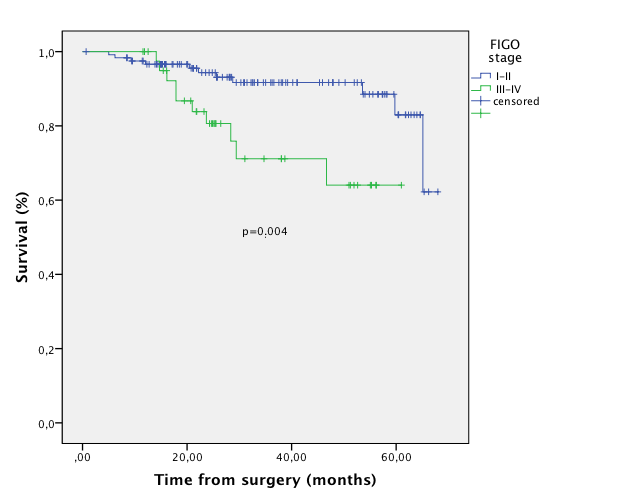

Supplement: Supplementary file 1 — Figure S1. Overall survival according to FIGO stage (n = 163). Kaplan-Meier survival analysis (p = 0.004 Log Rank, p = 0.013 Breslow test). (DOCX 64 kb) [file 12885_2018_5131_MOESM1_ESM.docx]

Supplementary Figure 8.ROC curves for ELR, ENLR, NLR and Platelet-to-Lymphocytes Ratio (PLR).


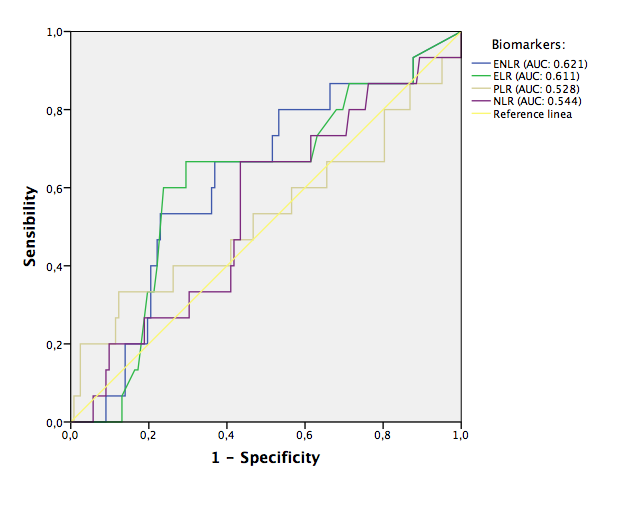

Supplement: Supplementary file 8 — Figure S8. ROC curves for ELR, ENLR, NLR and Platelet–to-Lymphocytes Ratio (PLR). (DOCX 81 kb) [file 12885_2018_5131_MOESM8_ESM.docx]
