# Supplementary material for: New pre-treatment eosinophil-related ratios as prognostic biomarkers for survival outcomes in endometrial cancer
Source: BMC Cancer. 2018 Dec 22;18:1280. doi: 10.1186/s12885-018-5131-x (PMC6304088; doi:10.1186/s12885-018-5131-x)

Supplementary Figure 2. Overall survival according to tumour grade (n=163). Kaplan-Meier survival analysis (p=0.001 Log Rank, p= 0.023 Breslow test).


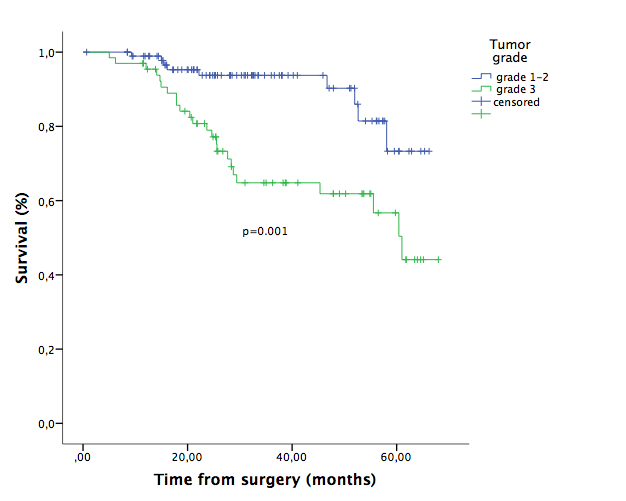

Supplement: Supplementary file 2 — Figure S2. Overall survival according to tumour grade (n = 163). Kaplan-Meier survival analysis (p = 0.001 Log Rank, p = 0.023 Breslow test). (DOCX 70 kb) [file 12885_2018_5131_MOESM2_ESM.docx]
