# Supplementary material for: New pre-treatment eosinophil-related ratios as prognostic biomarkers for survival outcomes in endometrial cancer
Source: BMC Cancer. 2018 Dec 22;18:1280. doi: 10.1186/s12885-018-5131-x (PMC6304088; doi:10.1186/s12885-018-5131-x)

Supplementary Figure 3. Overall survival according to ESMO-ESGO-ESTRO risk assessment (n=163): high-risk (groups 1-3) vs. low-risk (groups 4-6). Kaplan-Meier survival analysis (p=0.007 Log Rank, p= 0.005 Breslow test).


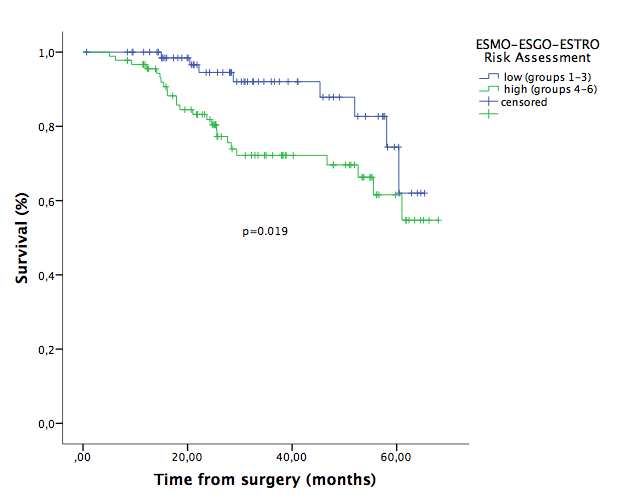

Supplement: Supplementary file 3 — Figure S3. Overall survival according to ESMO-ESGO-ESTRO risk assessment (n = 163): high-risk (groups 1–3) vs. low-risk (groups 4–6). Kaplan-Meier survival analysis (p = 0.007 Log Rank, p = 0.005 Breslow test). (DOCX 77 kb) [file 12885_2018_5131_MOESM3_ESM.docx]
