# Supplementary material for: New pre-treatment eosinophil-related ratios as prognostic biomarkers for survival outcomes in endometrial cancer
Source: BMC Cancer. 2018 Dec 22;18:1280. doi: 10.1186/s12885-018-5131-x (PMC6304088; doi:10.1186/s12885-018-5131-x)

Supplementary Figure 4. Overall survival according to tumour histology (n=163): endometrioid vs. non-endometrioid (p=0.001 Log Rank, p= 0.004 Breslow test).

.


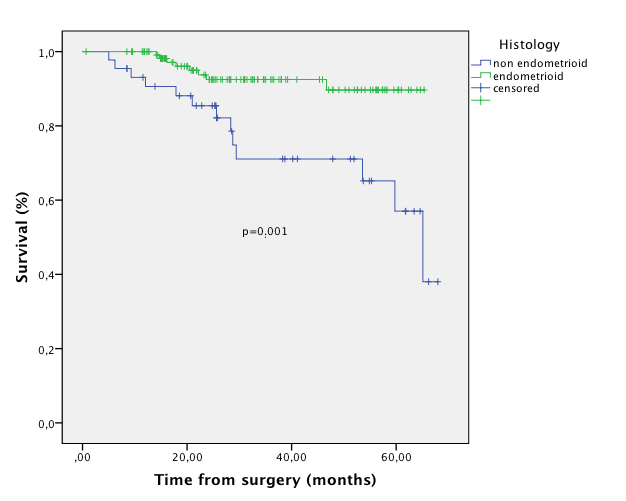

Supplement: Supplementary file 4 — Figure S4. Overall survival according to tumour histology (n = 163): endometrioid vs. non endometrioid (p = 0.001 Log Rank, p = 0.004 Breslow test). (DOCX 66 kb) [file 12885_2018_5131_MOESM4_ESM.docx]
