# Supplementary material for: New pre-treatment eosinophil-related ratios as prognostic biomarkers for survival outcomes in endometrial cancer
Source: BMC Cancer. 2018 Dec 22;18:1280. doi: 10.1186/s12885-018-5131-x (PMC6304088; doi:10.1186/s12885-018-5131-x)

Supplementary Figure 5: Overall survival according to patients’ age at diagnosis (n=163), p=0.047 Log Rank, p= 0.036 Breslow test.


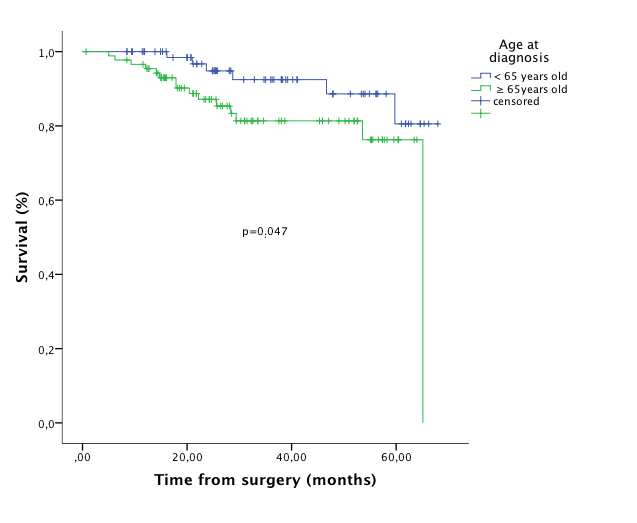

Supplement: Supplementary file 5 — Figure S5. Overall survival according to patients’ age at diagnosis (n = 163). Kaplan-Meier survival analysis (p = 0.047 Log Rank, p = 0.036 Breslow test). (DOCX 70 kb) [file 12885_2018_5131_MOESM5_ESM.docx]
