# Supplementary material for: New pre-treatment eosinophil-related ratios as prognostic biomarkers for survival outcomes in endometrial cancer
Source: BMC Cancer. 2018 Dec 22;18:1280. doi: 10.1186/s12885-018-5131-x (PMC6304088; doi:10.1186/s12885-018-5131-x)

Supplementary Figure 7. Overall survival for patients in High Risk (Risk groups 4-6 of ESMO 2015 classification) stratified by Eosinophil*Neutrophil-to-Lymphocytes Ratio (ENLR) according to cut-off 0.5.


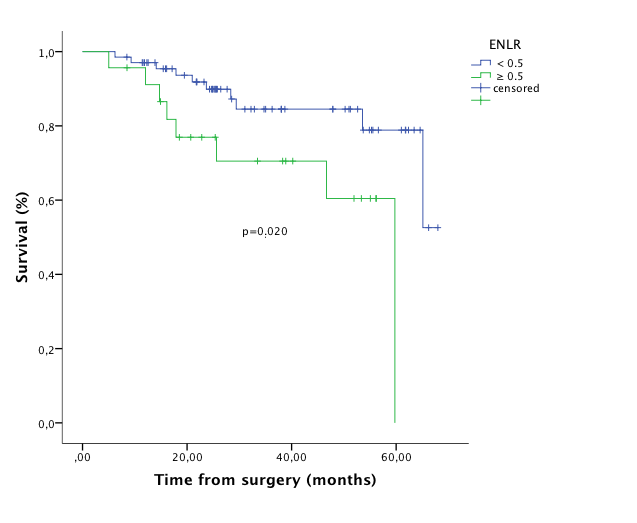

Supplement: Supplementary file 7 — Figure S7. Overall survival of patients in High Risk (Risk groups 4–6 of ESMO 2015 classification) stratified by Eosinophil*Neutrophil-to-Lymphocytes Ratio (ENLR) according to cut-off 0.5. (DOCX 65 kb) [file 12885_2018_5131_MOESM7_ESM.docx]
